# One‐Step Thermal Gradient‐ and Antisolvent‐Free Crystallization of All‐Inorganic Perovskites for Highly Efficient and Thermally Stable Solar Cells

**DOI:** 10.1002/advs.202202441

**Published:** 2022-06-19

**Authors:** Mahdi Malekshahi Byranvand, Tim Kodalle, Weiwei Zuo, Theresa Magorian Friedlmeier, Maged Abdelsamie, Kootak Hong, Waqas Zia, Shama Perween, Oliver Clemens, Carolin M. Sutter‐Fella, Michael Saliba

**Affiliations:** ^1^ Institute for Photovoltaics (ipv) University of Stuttgart Pfaffenwaldring 47 70569 Stuttgart Germany; ^2^ Helmholtz Young Investigator Group FRONTRUNNER IEK5‐Photovoltaik Forschungszentrum Jülich 52425 Jülich Germany; ^3^ Molecular Foundry Lawrence Berkeley National Laboratory 1 Cyclotron Road Berkeley CA 94720 USA; ^4^ Zentrum für Sonnenenergie‐ und Wasserstoff‐Forschung Baden‐Württemberg (ZSW) 70563 Stuttgart Germany; ^5^ Materials Sciences Division Lawrence Berkeley Laboratory 1 Cyclotron Road Berkeley CA 94720 USA; ^6^ Chemical Sciences Division Lawrence Berkeley National Laboratory 1 Cyclotron Road Berkeley CA 94720 USA; ^7^ Institute for Materials Science Chemical Materials Synthesis University of Stuttgart 70569 Stuttgart Germany

**Keywords:** all‐inorganic perovskites, crystallization, CsPbI_2_Br, in situ characterization

## Abstract

All‐inorganic perovskites have emerged as promising photovoltaic materials due to their superior thermal stability compared to their heat‐sensitive hybrid organic–inorganic counterparts. In particular, CsPbI_2_Br shows the highest potential for developing thermally‐stable perovskite solar cells (PSCs) among all‐inorganic compositions. However, controlling the crystallinity and morphology of all‐inorganic compositions is a significant challenge. Here, a simple, thermal gradient‐ and antisolvent‐free method is reported to control the crystallization of CsPbI_2_Br films. Optical in situ characterization is used to investigate the dynamic film formation during spin‐coating and annealing to understand and optimize the evolving film properties. This leads to high‐quality perovskite films with micrometer‐scale grain sizes with a noteworthy performance of 17% (≈16% stabilized), fill factor (FF) of 80.5%, and open‐circuit voltage (*V*_OC_) of 1.27 V. Moreover, excellent phase and thermal stability are demonstrated even after extreme thermal stressing at 300 °C.

## Graphical abstract

A spin‐forced (SF) method as a facile approach is proposed to control the crystallization kinetics of CsPbI_2_Br crystal growth, achieving a uniform, and high‐quality film. The perovskite film formation during spin‐coating and annealing is evaluated by optical in situ characterization. As a result, an efficiency of 17.0% with excellent thermal stability at 300 °C is achieved.

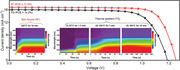

## 1 Introduction

Organic–inorganic metal halide perovskite solar cells (PSCs) have emerged as an attractive photovoltaic technology over the past decade achieving record power conversion efficiencies (PCEs) of up to 25.7%.^[^ ^1^, ^2^, ^3^ ^]^ However, the relatively poor long‐term stability of PSCs remains a significant barrier to large‐scale industrialization.^[^ ^4^, ^5^, ^6^, ^7^, ^8^ ^]^ Similar to other thin‐film solar cells, moisture, and oxygen instability could be minimized by device encapsulation^[^ ^9^, ^10^ ^]^ or improving the interfaces.^[^ ^11^ ^]^ However, the thermal stability of perovskite materials depends on the constituting components themselves. The most researched are hybrid organic–inorganic perovskites materials, which unfortunately contain volatile organic components such as methylammonium (MA) or formamidinium (FA). Thus, the thermal instability of hybrid PSCs remains an ongoing long‐term challenge in the quest of achieving stability.^[^ ^12^, ^13^, ^14^, ^15^, ^16^, ^17^, ^18^, ^19^ ^]^

As one response to these challenges, replacing organic with inorganic components has been reported.^[^ ^20^ ^]^ Especially, all‐inorganic cesium lead halide perovskites (CsPbX_3_, X = Cl, Br, I) have shown excellent thermal stability, where a stable perovskite phase can be retained even at temperatures exceeding 400 °C.^[^ ^21^, ^22^, ^23^ ^]^ CsPbI_3_ with a bandgap energy of 1.7 eV is a promising all‐inorganic perovskite composition not only for single‐junction solar cells but also for tandem applications, i.e., as the top cell combined with a low bandgap solar cell (*E*_gap_ ≈ 1.1 eV), such as crystalline silicon.^[^ ^24^ ^]^ However, the photoactive phase, *α*‐CsPbI_3_ (black, cubic) easily transforms to the photoinactive phase, *δ*‐CsPbI_3_ (yellow, orthorhombic), at room temperature or under trace moisture due to its low Goldschmidt tolerance factor (GTF) of 0.81.^[^ ^25^, ^26^ ^]^ This means that CsPbI_3_ exhibits the existence of multiple phases, which is known as polymorphism. On the other hand, CsPbBr_3_ is a wide bandgap perovskite (*E*_gap_ ≈ 2.3 eV) without any polymorphism. However, its wide bandgap entails a poor light absorption beyond 540 nm, rendering this material less relevant for single‐junction solar cells due to the resulting low short‐circuit current density.^[^ ^27^, ^28^ ^]^

In the trade‐off between low bandgap and phase stability, the most promising candidate is the mixed halide CsPbI_2_Br composition with a bandgap of *E*_gap_ ≈ 1.9 eV that still maintains phase‐stability (with a GTF of 0.84).^[^ ^29^, ^30^, ^31^ ^]^ However, only a uniform CsPbI_2_Br film with large crystalline grains and appropriate thickness is suited for high‐performance PSCs.^[^ ^32^, ^33^ ^]^ Moreover, the toxicity of *N*,*N*‐dimethylformamide (DMF), and chlorobenzene (CB) as the most common solvent and anti‐solvent for CsPbI_2_Br perovskite processing hinders the future practical industrial production of such all‐inorganic perovskite devices.^[^ ^34^, ^35^ ^]^ Besides the toxicity, the low solubility of inorganic precursors in DMF (below 0.5 m) limits perovskite film thicknesses to below 150 nm.^[^ ^21^ ^]^ Therefore, it is highly desirable to use a solvent with both low toxicity and high solubility to prepare high‐quality CsPbI_2_Br films for high‐performance all‐inorganic PSCs. The more environmentally friendly dimethyl sulfoxide (DMSO) with stronger polarity and coordination ability has frequently been used for increasing the CsPbI_2_Br precursor concentration up to 2 m.^[^ ^36^, ^37^ ^]^ However, the strong interaction of Lewis acid‐base PbI_2_‐DMSO adducts leads to more difficult solvent extraction from as‐deposited CsPbI_2_Br films during the crystallization process, resulting in a low‐quality film with abundant pin‐holes in bulk and at the surface.^[^ ^38^ ^]^ Thus, currently, there is no fast and straightforward method available for solution‐processed all‐organic perovskites with DMSO only. Understanding the fundamental nucleation kinetics and devising a technique to crystallize high‐quality perovskite films with DMSO is crucial to advancing the field.

A typically used method for CsPbI_2_Br film crystallization is thermal gradient (TG) annealing with a heat ramp from 50 to 160 °C.^[^ ^30^, ^31^, ^39^, ^40^, ^41^ ^]^ However, this method comprises many intermediate precursor states with different annealing temperatures, which according to the Ostwald rule these stages intrinsically hamper the final film quality, resulting in polymorphic, nonuniform film morphologies, and pin‐holes.^[^ ^41^ ^]^ Therefore, modifications have been applied to the TG method.^[^ ^40^, ^41^, ^42^, ^43^, ^44^, ^45^ ^]^ For example, recently, Chen et al. treated the perovskite films with isopropanol (IPA) as antisolvent before TG crystallization, consequently improving the CsPbI_2_Br film quality and device efficiency from 11.9% to 15.4%.^[^ ^41^ ^]^ Importantly, they demonstrated that the antisolvent treatment and TG annealing strongly affect the solvent extraction, i.e., DMSO extraction, from the wet precursor film and that high‐quality films require both methods.

Moreover, many different approaches such as room temperature solvent annealing,^[^ ^36^ ^]^ hot‐air‐assisted crystallization,^[^ ^46^, ^47^ ^]^ preheating the solution and substrate,^[^ ^33^ ^]^ and using mixed DMF/DMSO solvents^[^ ^38^, ^48^, ^49^ ^]^ have also been introduced to improve the crystallization. However, despite significant progress in this area, there is still a lack of a facile, environmentally friendly deposition method to further improve the crystallinity and morphology of CsPbI_2_Br films.

Here, we introduce a one‐step, thermal gradient‐, and antisolvent‐free method to precisely control the crystallization, i.e., nucleation and growth processes, achieving a homogenous, pinhole‐free perovskite film with large grain sizes. Using in situ optical characterizations, we propose that retained solvent after spin‐coating influences the film evolution and eventual quality during annealing. Guided by these insights, we optimize the formation process and validate it in complete solar cell devices. The n‐i‐p PSCs fabricated using our perovskite film showed a high *V*_OC_ of 1.27 V, FF of 80.5%, and 17% efficiency. Furthermore, our method results in CsPbI_2_Br perovskite films with excellent thermal stability at 300 °C.

## 2 Results and Discussion

**Figure 1:**
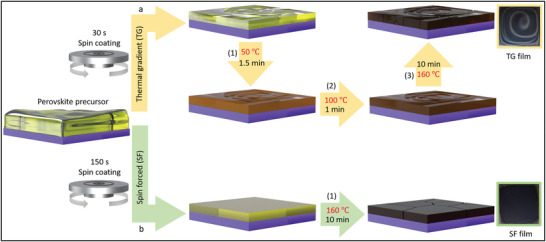
Schematic illustration of depositing the perovskite films by a) TG (conventional) and b) SF (introduced in this work) methods. Insets: photos of the SF and TG CsPbI_2_Br perovskite films.

We deposit CsPbI_2_Br perovskite films by spin‐coating the precursor solution containing stoichiometric PbI_2_, PbBr_2_, and CsI in DMSO solvent on indium tin oxide (ITO)‐covered glass substrates. For the TG method, i.e., conventional method, the perovskite precursor is spin‐coated for 30 s followed by a three‐step sequential thermal gradient annealing of 50 °C for 1.5 min, 100 °C for 1 min, and 160 °C for 10 min (see **Figure** **1**a). As reported in the literature, this annealing procedure is necessary to extract the residual DMSO molecules from the wet precursor film due to its high boiling point of 189 °C.^[^ ^41^ ^]^

However, even after precisely controlling these different annealing steps, a nonuniform perovskite film with a spiral‐shaped pattern formed (see the inset photo (top right) in Figure 1a). One possible mechanism for this spiral‐shaped morphology could be Rayleigh–Bénard convection, which develops various patterns and depends critically on the heating temperature.^[^ ^50^ ^],[^ ^51^ ^]^ Based on the classic Bénard cell pattern, the solution tends to flow upward during the annealing due to higher temperatures in the region close to the substrate. By reaching the hot solution to the top surface, its temperature decreases and it flows back to the bottom to get heated again, which could happen as convections frequently during the annealing time. These convections push each other in their surroundings due to the reverse circulation direction at the edge of the film, leading to formed polygon patterns in the solid films after annealing.^[^ ^52^ ^]^ In addition, we observe that transferring the wet precursor film (spin‐coated for 30 s) directly to the 160 °C hot plate without gradient steps leads to the formation of a *δ*‐phase CsPbI_2_Br film with an extremely rough surface and poor coverage due to fast and uncontrollable DMSO evaporation (Figure S1, Supporting Information).

To tackle this challenge, we introduce the spin‐forced (SF) method as a facile approach to control the crystallization kinetics (Figure 1b) by prolonging the spin‐coating time of the perovskite precursor to 150 s, followed by one‐step annealing at 160 °C for 10 min. As a result, we achieve a film with full coverage and a homogeneous black appearance (see the inset photo in Figure 1b (bottom right)).

**Figure 2:**
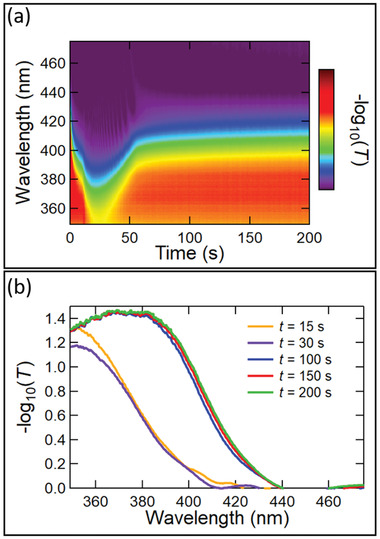
In situ UV–vis absorbance measurements of the wet perovskite films during spin‐coating (0–200 s). a) The contour plot of the film formation evolution, and b) line‐scans at selected points in time.

To analyze the film formation via the SF method, we use in situ UV–vis absorbance spectroscopy during spin‐coating and the subsequent annealing step to monitor the evolution of the absorbance spectra over time. To elucidate possible reasons for the improved quality of films prepared by the SF method, we monitored, in situ, the film over a total spin‐coating time of 200 s (**Figure** **2**) as well as drying during an additional 2500 s after spin‐coating at room temperature and inert atmosphere, i.e., inside the glovebox (Figure S2, Supporting Information). Figure 2a,b shows the contour plot and line scans of the absorbance evolution of the wet perovskite film during spin coating time, respectively. During the initial seconds of spin coating, we observe a reduction of the absorbance of the precursor solution due to solution ejection (i.e., loss of precursor material).^[^ ^53^, ^54^ ^]^ After this initial solution ejection, we observe a shift of the absorbance edge from about (400 ± 10) nm to (430 ± 10) nm within the first 30–40 s of spin‐coating, which we attribute to the removal of the bulk solvent and the formation of an intermediate precursor (IP) phase. Based on recent literature reports investigating hybrid perovskites, we speculate that the IP consists of a precursor‐solvent, i.e., a precursor‐DMSO, complex.^[^ ^55^ ^]^ Until the end of the spin‐coating step at 200 s, the absorbance edge of the precursor film stays almost unchanged. There is no indication of nucleation and/or crystal growth of the perovskite phase during the spin‐coating. However, as shown in Figure S2 (Supporting Information), about 1100 s after the end of the spin‐coating step (i.e., 1300 s after the start of the measurement), the absorbance edge shifts from about (430 ± 10) nm to (680 ± 10) nm at 1500 s indicating the crystallization of the perovskite phase. These results suggest that even the prolonged spin‐coating time in the SF method and consequent solvent extraction do not induce significant nucleation and/or growth of the perovskite phase during spin‐coating but rather homogenize the precursor film. However, we speculate that a longer spin‐coating time influences the amount of retained DMSO in the film, leading to different behavior and formation kinetics of the film during annealing.

**Figure 3:**
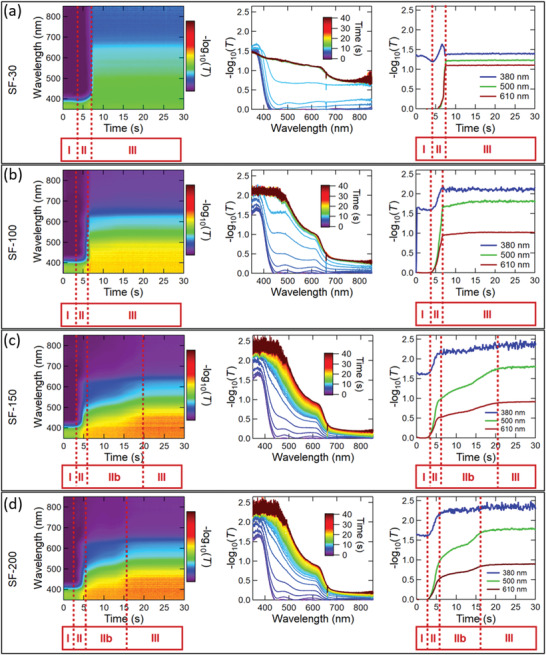
Evolution of the absorbance of the SF‐perovskite films during annealing of films prepared by different spin‐coating durations: a) SF‐30, b) SF‐100, c) SF‐150, and d) SF‐200. In each row, the left panel shows a contour plot of the film evolution, the middle panel shows line‐scans—evenly spaced in time versus wavelength, and the right panel shows line‐scans of different wavelengths over time. Note that no further changes were observed in the absorbance spectra for annealing times longer than 30 s.

To test this hypothesis, we carried out in situ UV–vis measurements during the subsequent annealing at 160 °C following the SF method. **Figure** **3**a–d shows the first 30 s of the 10 min annealing step for four different samples prepared using spin‐coating times of 30, 100, 150, and 200 s (generally called SF‐30, SF‐100, SF‐150, and SF‐200 in the following).

The film evolution during annealing can be separated into different stages (see Figure 3): During Stage I, the absorbance spectra stay mostly unchanged before the absorbance edge shifts to higher wavelengths during Stage II and finally remains unchanged again during Stage III. It can be seen that a prolonged spin‐coating duration leads to a faster evolution of the perovskite phase during the subsequent annealing (shorter Stages I and II in Figure 3), since the absorbance edge shifts faster to higher wavelengths (i.e., Stage II ends after 7s and 6s for the cases of SF‐30 and SF‐100, respectively, and after 5s for the cases of SF‐150 and SF‐200). This result indicates that the amount of retained DMSO in the film is lower for longer spin‐coating durations. Furthermore, a longer spin‐coating duration leads to a sharper absorbance edge. The sharpest absorbance edge, and therefore presumably the most ideal film properties, seem to be reached in the case of spin‐coating durations between SF‐150 and SF‐200 (see middle panels in Figure 3). Moreover, the evolution of the absorbance edge becomes more gradual for longer spin‐coating durations as can be seen by the presence of the “Transition Stage” IIb in Figure 3 for spin‐coating times of 150 and 200 s. In these samples, a rather slow shift of the absorbance edge follows the fast‐initial shift in Stage II. We attribute this slower shift to a possibly more controlled and more homogeneous crystal growth. These results support our hypothesis that a longer spin‐coating duration reduces the amount of retained DMSO in the film due to a slower, more controlled extraction during the prolonged spin‐coating step compared to the rather quickly induced extraction during the annealing step.

This development might be accompanied by a more homogeneous distribution of colloids in the wet films after longer spin‐coating times. In conclusion, a longer spin‐coating time enables better control and likely more homogeneous nucleation and growth of the perovskite films, thereby resulting in higher quality CsPbI_2_Br films as judged by their absorbance characteristics and discussed later based on scanning electron microscopy (SEM) images. Interestingly, the Transition Stage IIb is shorter in the case of the SF‐200 film compared to the SF‐150 film, indicating an optimal spin‐coating time of 150 s. This result suggests that there is an optimal amount of remaining DMSO in the wet film, which possibly facilitates diffusion during the annealing step. This interpretation is consistent with previous reports that too slow solvent‐extraction can lead to a high density of nuclei with heterogeneous size distribution and therefore reduce the density of the films and the homogeneity of the grain sizes.^[^ ^56^, ^57^ ^]^

Considering the tunability of our SF method, we measured the in situ absorbance for various combinations of precursor concentration and spin rate as well. As shown in Figure S3 (Supporting Information), although the relation between the deposition parameters is complicated, there is a prominent trend in becoming the process window wider for higher concentrations (within the probed regions of the parameter space). While no combination of parameters leads to an optimal evolution, there are two and three conditions with (near to) optimal results for concentrations of 1.3 and 1.5 m, respectively. Generally speaking, it seems that rather high spin rates and spin‐coating durations are required to achieve high‐quality films.

Moreover, we investigated the effect of different annealing temperatures of 100 °C, 160 °C, and 200 °C on the perovskite film formation at the optimized spin‐coating conditions (3000 rpm, 150 s) and precursor concentration (1.3 m). As shown in Figure S4 (Supporting Information), among different annealing temperatures, the best quality of perovskite film was only achieved for 160 °C, presenting the intermediate phase IIb during annealing with a sharp absorbance edge.

To evaluate the crystal quality, we systematically investigated the crystallinity of annealed CsPbI_2_Br perovskite films with different spin‐coating times utilizing X‐ray diffraction (XRD) characterization (Figure S5, Supporting Information). Among the different samples, the final SF‐150 perovskite film showed the highest peak intensities, suggesting the highest crystallinity and phase purity.

**Figure 4:**
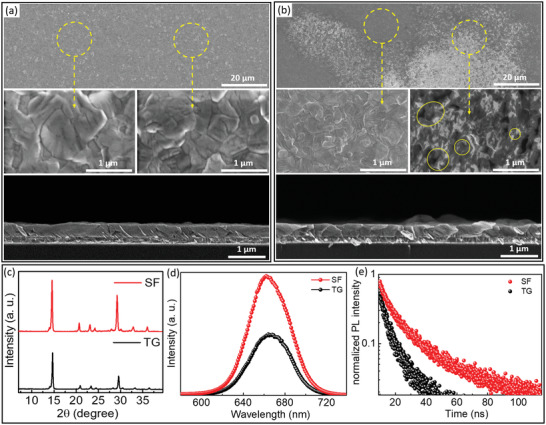
Top‐view and cross‐sectional SEM images of a) SF‐ and b) TG‐films. c) XRD patterns, d) steady‐state photoluminescence (SSPL), and e) time‐resolved PL (TRPL) from SF‐ and TG‐films.

We recorded top view and cross‐sectional SEM images to resolve the perovskite grain sizes and film morphologies (**Figure** **4**a,b). The SF‐150 film shows homogeneous morphology in the whole area with uniform grain sizes (see the low‐ and high‐magnification top‐view SEM images in Figure 4a). Moreover, the cross‐sectional image confirms a compact film morphology, facilitating a good contact with the hole transport layer (HTL) after device fabrication. Interestingly, the grain sizes uniformly expand to about 1 µm, which is significantly larger than the film thickness (≈450 nm) with a reduced density of grain boundaries (GBs). Figure S6 (Supporting Information) compares the morphology of this film with the SF‐100 and SF‐200 films. The SF‐100 film shows much smaller grain sizes and a higher density of GBs than the SF‐150 film (Figure 4a), emphasizing that carefully controlling the amount of residual DMSO in the IP film during spin‐coating enabled us to achieve high‐quality CsPbI_2_Br films. As demonstrated in Figure S6b (Supporting Information), the SF‐200 perovskite film also shows bigger grain sizes than the 100 s film, but (in agreement with the abovementioned hypothesis) some nonuniformities can be observed in the cross‐sectional image compared to the SF‐150 film (Figure 4a). Possible reasons for this inferior morphology include a broad size distribution of the perovskite nuclei due to the very long DMSO extraction during spin‐coating.^[^ ^56^ ^]^ Additionally, the diffusion of the constituent elements during annealing might be more limited if too much DMSO is already extracted during spin‐coating, thereby limiting grain growth.

In agreement with the previous discussion, we, therefore, propose that there is an optimum amount of DMSO to extract during spin‐coating to control the density of nucleation sites for the subsequent annealing step. In the case of short spin‐coating times, rather high amounts of DMSO remain in the IP films, leading to a small number of nucleation sites, which then grow heterogeneously and a coarse morphology results.^[^ ^56^, ^57^ ^]^ Furthermore, a rather high amount of DMSO is extracted during annealing, which might further facilitate inhomogeneous growth. In the case of longer spin‐coating times, the amount of DMSO is reduced, leading to a higher density and narrower size distribution of nucleation sites^[^ ^57^ ^]^ and, therefore, better film morphology finally. If, however, a critical spin‐coating time is exceeded, i.e., the amount of remaining DMSO in the film is too low at the beginning of the annealing step, the ability of the precursor clusters to rearrange and to induce a high number of homogeneously sized nucleation sites will be limited and ultimately results in lower crystal quality and nonuniformities as well. Therefore, we select the 150 s spin‐coating time as the optimum to achieve the highest quality SF film, generally called SF‐film in the following, and compare it to the CsPbI_2_Br perovskite film fabricated by the conventional TG method film. As shown in the low‐ and high‐magnification top‐view SEM images in Figure 4b, the TG film comprises two areas with compact morphology but polydisperse small grain sizes of 200–500 nm and disordered morphology with numerous pinholes. Moreover, the cross‐sectional SEM image shows a nonuniform film with different thicknesses in different regions and a high density of GBs, providing poor contact with the HTL.

To understand the origin of the poor morphology of the TG‐film, we explore the crystallization from the IP phase to nucleation during each annealing step of the TG method using in situ absorption spectroscopy. Figure S7 (Supporting Information) shows contour plots of the absorbance spectra of IP films during annealing following the TG method. Compared to the evolution of the absorbance spectra of the SF‐films shown in Figure 3, the development of the perovskite‐related absorbance edge around (670 ± 10) nm is delayed for the TG method. The shift of the IP‐related absorbance edge from about (400 ± 10) nm to (430 ± 10) nm that was observed during the prolonged spin‐coating process in the SF method takes place between 0 and 30 s during the first annealing step (1) of the TG method, i.e., 50 °C for the 90s (Figure S7, Supporting Information). Furthermore, the transition from IP to perovskite film occurs throughout the remainder of the first and almost the complete second annealing step (2), i.e., 100 °C for 60s, which is much slower than in the case of the SF method. We attribute this delay partially to the shorter spin‐coating duration and partially to the lower temperatures utilized for the annealing. It leads to a perovskite film with nonuniform morphology in different areas after the final annealing step at the higher temperature of 160 °C for 10 min (Figure S8, Supporting Information). Therefore, we propose that after very short spin‐coating and during low‐temperature annealing, i.e., 50 and 100 °C, the DMSO extraction is strongly delayed compared to the SF case, leading to more shrinkage of the film during annealing as well as a less controlled crystallization due to the higher crystallization barrier.

Next, we explore the crystallinity of the perovskite films prepared by TG and SF methods using XRD characterization. Figure 4c shows the cubic *α*‐phase of CsPbI_2_Br with preferable crystal growth along the (100) facet without any trace of unfavorable *δ*‐phase for both perovskite films. However, the SF‐film shows almost twice as high diffraction peak intensities than the TG‐film, again suggesting enhanced crystallinity. This result is consistent with the denser, more homogeneous, and larger grain sizes of the SF‐film. Figure S9 (Supporting Information) shows normalized UV–vis absorbance spectra of TG and SF films. The SF‐film exhibits slightly higher absorbance values than the TG‐film due to compact morphology and enlarged grain sizes with better crystallinity.

Additionally, we study the quality of the perovskite films by investigating the dynamics of charge carrier recombination via steady‐state photoluminescence (SSPL) and time‐resolved PL (TRPL) measurements. For this, we prepared the perovskite samples on bare glass substrates to rule out the charge extraction ability of the tin oxide (SnO_2_) layer as an electron transport layer (ETL). As shown in Figure 4d, the PL intensity of the SF‐film increases approximately by a factor of 2 compared to the TG‐film, demonstrating a dramatically decreased nonradiative recombination in the SF‐film due to better film quality with the lower density of defect states in the bandgap.^[^ ^58^, ^59^ ^]^ Additionally, Figure S10 (Supporting Information) compares the SSPL of different SF‐films, confirming the better quality of the SF‐150 film, which also shows the highest PL intensity compared to the other SF‐films. To gain quantitative information about this behavior, we performed TRPL measurements (Figure 4e). We fit the decay curves with a biexponential function, including short lifetime (*τ*1) and long lifetime (*τ*2) factors, which represent nonradiative surface and bulk recombination, respectively (see Table S1, Supporting Information). The SF‐film shows greatly improved surface and bulk recombination lifetimes of 3.3 and 14.3 ns compared to lower lifetimes of 1.9 and 8.2 ns for TG‐film. This improvement is attributed to the reduced intrinsic defect density in the SF‐film due to its compact crystals, large grain sizes, and lower density of grain boundaries, which reduce the nonradiative recombination both at the interfaces and in the bulk of the film.

**Figure 5:**
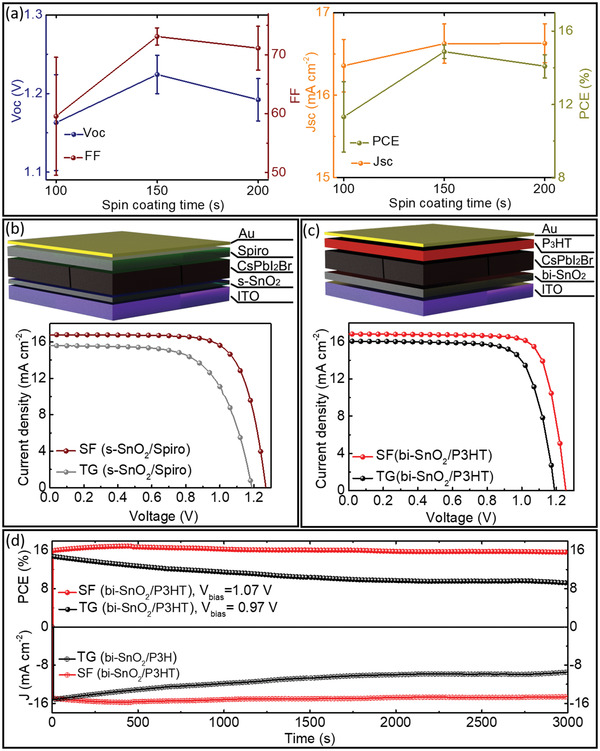
a) Photovoltaic parameters (*V*_OC_, FF, *J*_SC,_ and PCE) extracted from *J*–*V* measurement of 30 PSCs fabricated with SF‐30, SF‐100, SF‐150, and SF‐200 films (fabricated with s‐SnO_2_ as ETL and Spiro‐OMeTAD as HTL). The schematic illustrations of device structures and the champion *J*–*V* characteristics of fabricated TG and SF‐PSCs with b) s‐SnO_2_ and Spiro‐OMeTAD, c) bi‐SnO_2_ and P3HT under standard test conditions (100 mW cm^−2^ intensity, AM1.5G spectrum, and 25 °C device temperature). d) The stabilized power output (top) and current (bottom) of TG‐ and SF‐PSCs at the maximum power point voltage over time.

To confirm the quality of different CsPbI_2_Br films, we directly fabricated conventional n‐i‐p PSCs on ITO‐covered glass, with a single SnO_2_ layer as ETL (s‐SnO_2_), CsPbI_2_Br perovskite as the photoactive layer, 2,2′,7,7′‐Tetrakis(*N*,*N*′‐di‐pmethoxyphenylamine)‐9,9′‐spirobifluorene (Spiro‐OMeTAD) as HTL, and gold as the metal back contact (see the Experimental Section). First, we compare the photovoltaic (PV) parameters of fabricated SF‐PSCs with different spin‐coating times. As shown in **Figure** **5**a, the PV parameters gradually increase with the spin‐coating time from 100 to 150 s, i.e., SF‐100 to SF‐150, which confirms the improved perovskite film quality. However, the PV parameters decreased for PSCs fabricated with SF‐200 films, indicating lower film quality than the SF‐150 films and in line with the hypothesis described above.

Therefore, we again compare the PV parameters of the optimum SF‐PSCs, i.e., SF‐150 film, with TG‐PSCs to understand better the film quality's effect on the device performance. Figure S11 (Supporting Information) illustrates the statistical data of photovoltaic parameters of 30 SF‐ and TG‐PSCs. Remarkably, the SF‐PSCs show significantly improved device performance and reproducibility compared to PSCs prepared by the TG method. The TG‐PSCs show an average *PCE* of (10.0 ± 1.8)%, with an average *J*_SC_ = (15.3 ± 0.4) mA cm^−2^, *V*_OC_ = (1.16 ± 0.04) V, and *FF* = (56.1 ± 9.0)%. Remarkably, for the SF films the average *PCE* increases to (14.9 ± 0.4)%, with an average *J*_SC_ = (16.6 ± 0.2) mA cm^−2^, *V*_OC_ = (1.23 ± 0.02) V, and *FF* = (73.1 ± 1.4)%.

Figure 5b presents reverse scan direction *J*–*V* characteristics of the champion SF‐ and TG‐PSCs. We observed a substantial performance improvement from 12.0% for TG to 15.6% for SF PSC, which is reflected in the values for all the PV parameters (**Table** **1**). The *J*_SC_ and *FF* improved from 15.6 to 16.7 mA cm^−2^ and 65.0% to 73.9%, respectively, for SF‐PSC compared to TG PSC, mainly due to higher absorbance ability, better interface with HTL due to enlarged crystal size, and a more compact SF film. We also measured the external quantum efficiency (EQE) of the champion PSCs and used that data to calculate *J*_SC_ values for both PSCs. As shown in Figure S12 (Supporting Information), the SF PSC achieved much higher EQE values than the TG PSC, with calculated *J*_SC_ values of 14.7 and 16.1 mA cm^−2^, respectively. These values are in good agreement with the values achieved from the corresponding *J–V* curves from these two procedures (with an acceptable error of 5%).^[^ ^60^ ^]^

**Table 1:** Photovoltaic parameters obtained from the champion SF‐and TG‐PSCs fabricated with different ETLs and HTLs

PSCs	PCE [%]	*J*_SC_ [mA cm^−2^]	FF [%]	*V*_OC_ [V]
TG (s‐SnO_2_/Spiro)	12.0	15.6	65.0	1.18
SF (s‐SnO_2_/Spiro)	15.6	16.7	73.9	1.26
TG (bi‐SnO_2_/P3HT)	14.2	16.0	74.2	1.19
SF (bi‐SnO_2_/P3HT)	17.0	16.6	80.5	1.27

The recent development of CsPbI_2_Br PSCs demonstrated that the use of the bilayer SnO_2_ (bi‐SnO_2_) as ETL^[^ ^61^ ^]^ and Poly(3‐hexylthiophene) (P3HT) as HTL^[^ ^31^, ^61^ ^]^ can improve the photovoltaic parameters significantly. Therefore, we fabricated PSCs with bi‐SnO_2_ and P3HT to demonstrate the versatility of our perovskite crystallization method and reach higher PCEs. As shown in Figure 5c and Table 1, the champion SF‐PSC with this structure showed a high PCE of 17% with an excellent FF of 80.5% and *V*_OC_ of 1.27 V, which is only ≈0.4% PCE lower than the highest reported PCE of 17.46% (see Table S3, Supporting Information).^[^ ^46^ ^]^ Although the achieved *J*_SC_ and FF for SF‐PSCs are considerable, we believe the *V*_OC_ still can be improved considering the wide bandgap of CsPbI_2_Br (≈1.9 eV) by other strategies such as interface modification,^[^ ^62^ ^]^ surface passivation,^[^ ^63^ ^]^ composition engineering,^[^ ^30^, ^46^ ^]^ and novel charge extraction layers.^[^ ^64^ ^]^

Nevertheless, similar to s‐SnO_2_/Spiro device structure, the TG method leads to a lower PCE of 14.2% due to lower PV parameters than SF‐PSC (Table 1). The statistical data of photovoltaic parameters confirmed the reproducibility of our method (Figure S13, Supporting Information).

The wide bandgap of all‐inorganic PSCs provides an excellent potential to deliver high voltages for tandem application.^[^ ^65^ ^]^ However, these perovskite materials typically show numerous defect states in their structures, mainly due to poor crystallization, leading to high nonradiative recombination and high *V*_OC_ deficit characteristics (defined as the difference between the absorber bandgap and the measured *V*_OC_).^[^ ^66^ ^]^ We achieve an outstanding *V*_OC_ of 1.27 V for the SF‐film compared to 1.19 V for the TG‐film for fabricated PSCs with bi‐SnO_2_ as ETL and P3HT as HTL, implying lower nonradiative recombination losses due to improved perovskite crystallinity and decreased grain boundaries as demonstrated by PL spectroscopy (see Figure 4d,e). Furthermore, to obtain indications of the relevant recombination mechanism at the perovskite/HTL, we measured the ideality factor of fabricated PSCs, which is determined by the *V*_OC_ dependence on the light intensity. The diode ideality factor *n*_id_, deduced from the slope described by *n*_id_ *k*_B_ *T*/*q*, where *k*_B_ is the Boltzmann constant, and *T* is temperature, where 1.78 and 1.29 are achieved for the TG and the SF PSCs, respectively (Figure S14, Supporting Information). This further indicates reduced surface defects on the perovskite/HTL interface in SF PSC (with a value closer to 1), which is in good agreement with the PL data.

Moreover, as shown in Figure S15 (Supporting Information), the SF PSC shows a negligible *J–V* hysteresis as small as ≈1% PCE difference compared to a high value of ≈3% for the TG‐PSC, which is likely due to the overall reduced bulk defect density and suppressed recombination during SF‐PSC operation with better film quality.^[^ ^40^, ^67^, ^68^ ^]^ To better understand the reduced hysteresis and verify the *PCE* of PSCs under stable operation conditions, we determine the stabilized power output (SPO) of different PSCs by measuring the current at a fixed maximum power point voltage over time.^[^ ^69^ ^]^ As shown in Figure 5d, the SF‐PSC shows an excellent stabilized *PCE* of ≈16% with a stable current density of ≈15 mA cm^−2^ under a constant bias voltage of 1.07 V for 3000 s, very close to those obtained from the *J–V* curves under both forward and reverse scan directions (Figure 5b and Table 1). In contrast, the TG‐PSC shows only ≈9% stabilized *PCE* under a constant bias voltage of 0.97 V with an unstable current density of ≈9.4 mA cm^−2^, which confirms the observed high *J–V* hysteresis originating from low‐quality TG‐film.

**Figure 6:**
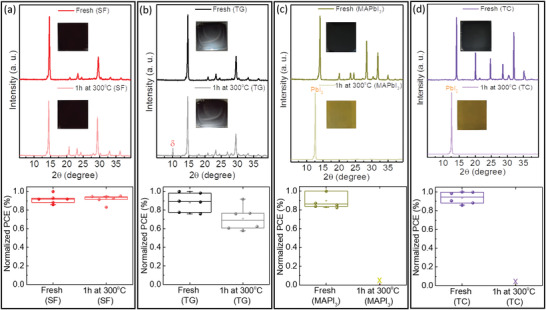
The XRD patterns and normalized PCEs of a) SF‐, b) TG‐, c) MAPbI_3_‐, d) TC‐films, before and after heat treatment at 300 °C for 1 h. Insets: photos of the appearance of different perovskite films.

Perovskite materials and fabricated devices should be stable at 85 °C under standard^[^ ^74^ ^]^ testing conditions.^[^ ^70^ ^]^ However, higher stability over 85 °C also would be attractive for other PSCs applications such as concentrated or space photovoltaics.^[^ ^71^ ^]^ For this purpose, we compare the thermal stability of inorganic and organic–inorganic perovskite films by extreme heating to 300 °C for 1 h under an inert atmosphere, exceeding even the most aggressive standard solar cell operating temperatures at 85 °C (**Figure** **6**a–d).

First, we compare the thermal stability of fabricated all‐inorganic perovskite films with different methods. Remarkably, the SF‐film shows excellent phase stability after heat treatment, with only the *α*‐phase remaining and without any trace of *δ*‐phase for the CsPbI_2_Br perovskite film (see the XRD pattern in Figure 6a). Moreover, the appearance of the perovskite films before and after heat treatment remained black. In addition, the fabricated PSCs (6 independent solar cells) with fresh and heat‐treated SF‐films show similar efficiencies, demonstrating the high thermal stability of the SF‐film due to its high quality (see the PV data in Figure 6a). In contrast, as shown in Figure 6b (see the XRD pattern), a small *δ*‐phase peak appears in the XRD data after heat treatment of the TG‐film at 300 °C, which could be related to the lower quality of this film due to the nonuniform morphology in the different areas (see top‐view SEM images in Figure 4b). As a result, this partial phase transition in the heat‐treated film leads to ≈10% relative lower PCEs compared to fresh TG‐film (see the PV data in Figure 6b).

Second, we compared the thermal stability of all‐inorganic films with the most widely used organic–inorganic perovskite films, i.e., MAPbI_3_ and triple cation (TC) compositions of Cs_0.5_(MA_0.17_FA_0.83_)_0.95_Pb(I_0.83_Br_0.17_).^[^ ^20^ ^]^ Unlike the all‐inorganic perovskites, i.e., SF‐ and TG‐films, the black color of both MAPbI_3_ and TC films converted to yellow after the 300 °C heat treatment (see the inset photographs in Figure 6c,d). The XRD patterns confirm that both MAPbI_3_‐ and TC‐films completely degraded as the perovskite peaks seen in patterns of the fresh films vanish while PbI_2_ peaks appear in the heat‐treated films, suggesting the complete evaporation of the organic component in these films (Figure 6c,d). The heat‐treated MAPbI_3_‐ and TC‐films, since they had turned yellow and degraded, were obviously unsuited for further consideration as solar cells (see PV data in Figure 6c,d). This result suggests that first, substituting the organic component to inorganic counterparts in perovskite films, i.e., all‐inorganic perovskites, and, second, improving the quality of these films by developing precise crystallization methods, i.e., SF method, does deliver heat‐resilient films for long‐term stability. Moreover, we also characterized the thermal stability of the fabricated PSCs with the SF method under standard 85 °C conditions. As shown in Figure S16 (Supporting Information), the PCEs of solar cells remained ≈85% of their initial values after aging under 85 °C in an N_2_ atmosphere for 120 h.

We also examine the moisture stability of different CsPbI_2_Br films and their devices by exposing them to the high humidity of ≈60% at room temperature. As shown in the XRD patterns in Figure S17a,b (Supporting Information), the *δ*‐phase peak appeared for both SF‐ and TG‐films after 15 min under this high humidity condition. However, the intensity of the *δ*‐phase peak for the SF film was much lower than the TG sample, implying a slower conversion to the *δ*‐phase due to higher film quality (see the inset photos in Figure S17a,b, Supporting Information). The fabricated PSCs with these films also show the same behavior, with remaining average PCEs of ≈50% for SF‐PSCs compared to ≈20% for TG‐PSCs (see the normalized PCE in Figure S17a,b, Supporting Information). However, both films became completely yellow upon extending the exposure time to 30 min, resulting in a total conversion of the *α*‐phase to the *δ*‐phase (see the XRD patterns, inset photographs and *PCEs* for 30 min in Figure S17a,b, Supporting Information). Accordingly, we could not fabricate functioning devices from fully degraded SF and TG films (Figure S17a,b, Supporting Information). This poor moisture stability is consistent with previous reports about CsPbI_2_Br films under severe humidity conditions.^[^ ^41^ ^]^ At the same time, it shows that the improved film morphology of the SF method improves the moisture stability compared to the TG method due to fewer grain boundaries where moisture can penetrate.^[^ ^72^ ^]^ Additionally, the UV‐light stability of SF‐PSCs was also investigated. As shown in Figure S18 (Supporting Information), the PCEs of unencapsulated solar cells could maintain ≈80% of their initial values after aging under UV irradiation in an N_2_ atmosphere for 130 h. We attribute the UV stability of PSCs most likely to the high quality of SF‐CsPbI_2_Br films with minimum defect density in bulk.

## 3 Conclusions

In summary, we introduce a simple one‐step thermal gradient‐ and antisolvent‐free method for precisely controlling the crystallization and morphology of the CsPbI_2_Br films. We find out that the film quality and phase transition are highly dependent on the spin‐coating time of the perovskite precursor before the annealing step. The in situ UV–vis absorbance confirms that extending the spin‐coating duration to 150 s reduces the amount of retained DMSO in the film, leading to a more homogeneous distribution of colloids in the IP films, which in turn leads to homogeneous nucleation and growth of the perovskite films and thus improved film quality. We obtain a uniform and compact perovskite *α*‐CsPbI_2_Br film with large grain sizes and improved grain boundaries by the SF method. We also compare our method with TG as the conventional deposition method.

Moreover, we test the different perovskite films with different ETLs and HTLs to achieve the best PCEs. The PSC based on this SF film (fabricated with bi‐SnO_2_ as ETL and P3HT as HTL) demonstrates a high PCE of 17% (stabilized efficiency of ≈16%) with an excellent *V*_OC_ of 1.27 V and FF of 80.5%, as well as, negligible hysteresis. Low defect density as another indication of high film quality was demonstrated by PL spectroscopy. In contrast, the PSC based on TG films with a similar device structure only delivers a champion *PCE* of 14.2% (stabilized efficiency of ≈9%) with more significant hysteresis due to lower film quality. In addition, SF‐films show excellent thermal stability after exposure to 300 °C as compared to TG‐films, a result which we can directly relate to their high crystallinity and better film quality. We believe our novel method can facilitate high‐quality CsPbI_2_Br films, leading to highly efficient all‐inorganic PSCs. Finally, the SF method could be applicable for fabricating other perovskite compositions.

## Conflict of Interest

The authors declare no conflict of interest.

## Supporting information

Supporting Information

## Data Availability

The data that support the findings of this study are available from the corresponding author upon reasonable request.
