# Supplementary material for: One‐Step Thermal Gradient‐ and Antisolvent‐Free Crystallization of All‐Inorganic Perovskites for Highly Efficient and Thermally Stable Solar Cells
Source: Adv Sci (Weinh). 2022 Jun 19;9(23):2202441. doi: 10.1002/advs.202202441 (PMC9376821; doi:10.1002/advs.202202441)
Supplement: Supplementary file 1 — Supporting Information [file ADVS-9-2202441-s001.pdf]

Supplemental Information

One-Step Thermal Gradient- and Antisolvent-Free Crystallization of All-Inorganic Perovskites for Highly Efficient and Thermally Stable Solar Cells

Mahdi Malekshahi Byranvand,* Tim Kodalle, Weiwei Zuo, Theresa Magorian Friedlmeier, Maged Abdelsamie, Kootak Hong, Waqas Zia, Shama Perween, Oliver Clemens, Carolin M. Sutter-Fella, and Michael Saliba*

Experimental Section

Device fabrication

The all-inorganic perovskite solar cells (PSCs) were fabricated in n-i-p solar cell architecture: glass substrate, indium tin oxide (ITO)/nanoparticle-based SnO_2 electron transport layer (ETL)/ CsPbI2Br perovskite absorber layer/2,2′,7,7′-tetrakis(N, N′-di-p-methoxy phenylamine)-9,9′-spirobifluorene (SpiroOMeTAD) hole transport layer (HTL)/silver back electrode.

The pre-patterned ITO-on-glass substrates (sheet resistance 15 Ω sq−1, Luminescence Technology) were cleaned consecutively with deionized (DI) water, acetone (Ac, Sigma-Aldrich), and isopropyl alcohol (IPA, Sigma-Aldrich) in an ultrasonic bath for 10 min, followed by UV-O_3 treatment for 30 min. In the next step, a thin layer of 2.04 wt% colloidal SnO2 nanoparticles (Alfa Aesar, 15 wt% aqueous solutions) was spin-coated on ITO substrates (4000 rpm for 30 s) as ETL, followed by an annealing step at 150 °C for 30 min. For the bilayer SnO_2 (bi-SnO_2), the 4 days aged $SnCl_2$ solution (Sigma-Aldrich, 0.189 g $SnCl_2$ in 10 ml EtOH and 36 µl DI H2O) was deposited on already prepared single layer SnO_2 (s-SnO_2) at 6000 rpm for 30 s, followed by continuous baking at 100 ^{o}C for 10 min and 180 ^{o}C for 1 hour. The perovskite

absorber CsPbI2Br films were deposited by different methods on top of the preheated SnO_2 ETL substrates (45 ^oC, 1 min) via spin-coating the precursor solution containing stoichiometric PbI_2, $PbBr_2$, and CsI in DMSO. The precursor solution concentration was 1.3 M, if not mentioned otherwise.

In the thermal gradient (TG) method, the perovskite precursor solution was spin-coated on substrates for 30 s followed by three steps sequential thermal gradient annealing of 50 ^oC for 90s, 100 °C for 60s and 160 °C for 10 min. In the spin forced (SF) method, the spin-coating time of the perovskite precursor was prolonged to 30 s, 100 s, 150 s, and 200 s, followed by one step annealing at 160 °C for 10 min. The rotation speed was set to 3000 rpm for both methods if not mentioned otherwise. Afterward, the Spiro-OMeTAD solution as HTL was spin-coated on top of the different perovskite films at 4000 rpm for 30 s. The Spiro-OMeTAD precursor solution was prepared by dissolving 92 mg Spiro-MeOTAD (Luminescence Technology) in 1 mL chlorobenzene (CB, Sigma-Aldrich) with the additives 21 µL lithium bis(trifluoromethanesulfonyl) imide stock solution (520 mg Li-TFSI, Sigma-Aldrich, in 1 ml Acetonitrile), 28.5 µL 4-tert-butylpyridine (TBP, Sigma-Aldrich) and 15 µL stock solution cobalt(III) tris(bis(trifluoromethylsulfonyl)imide) (31 mg FK209, Dyesol, in 84 µL Acetonitrile). The P3HT solution precursor solution was prepared by dissolving 15 mg in 1 ml CB with the additives 45 µL Li-TFSI stock solution (10 mg Li-TFSI in 1 ml Acetonitrile) and 10 µL TBP. The P3HT solution as was spin-coated on top of the different perovskite films at 2500 rpm for 25 s. Finally, 80 nm gold electrode was deposited by thermal evaporation through a shadow mask to define the active area to 0.16 cm^2.

Characterization

The solar cells were characterized using a 21 Channel LED Solar Simulator (Wavelabs Solar Metrology Systems) with an AM1.5G spectrum (100 mW cm^{-2}). The *J–V* characteristics were measured in both reverse and forward direction with a constant scan rate of circa 10 mV s^{-1} (Keithley 2400 source measurement unit). The stabilized power output (SPO) of different PSCs was determined by measuring the current at a fixed maximum power point voltage over time. The UV-Vis absorbance spectra of the perovskite films were measured with a PerkinElmer LAMBDA 1050. For the *in situ* absorbance measurements, an Ocean Optics spectrometer (Flame) coupled with fiber optics was used to acquire the transmission measurements with an integration time of about 0.1 s to 0.25 s per transmission spectrum and a wavelength spacing of about 1.3 nm. The equation [$A\lambda = -\log_{10}(T\lambda)$] was used to calculate the UV-Vis absorption spectra from the transmission spectra, where $A\lambda$ is the absorbance at a certain wavelength (λ), and $T\lambda$ is the corresponding transmitted radiation. The *in situ* UV-Vis transmission/absorption measurements during spin-coating were performed using a set-up described in the previous work.[1] The In-situ UV-Vis transmission/absorption measurements during thermal annealing were performed using a custom-built heating stage with a hole that allows transmission analogous to the one described in previous work.[1] Steady-state photoluminescence (SSPL) measurements were conducted using a room temperature solid-state photoluminescence (RT-PL), FluoroMax Plus HORIBA Scientific. Time-resolved photoluminescence (TRPL) measurements were performed using a pulsed laser source with a wavelength of 474 nm and a fluence of ~ 400 nJ/cm^2. The samples were excited from the glass side under ambient conditions. The perovskite films crystallinity was examined by an X-Ray diffraction (XRD, SmartLab SE from Rigaku) machine with a HyPix-400 (2D HPAD) detector. The SEM images were obtained

with a Zeiss Crossbeam 550 scanning electron microscope with a low acceleration voltage of 2 kV and a low beam current of 42 pA (ZSW). The thermal stability test from perovskite film was performed inside the glove box under the N_2 atmosphere. For the humidity test, perovskite films and PSCs were kept in an ambient atmosphere (relative humidity ~60%).

Supporting figures and tables

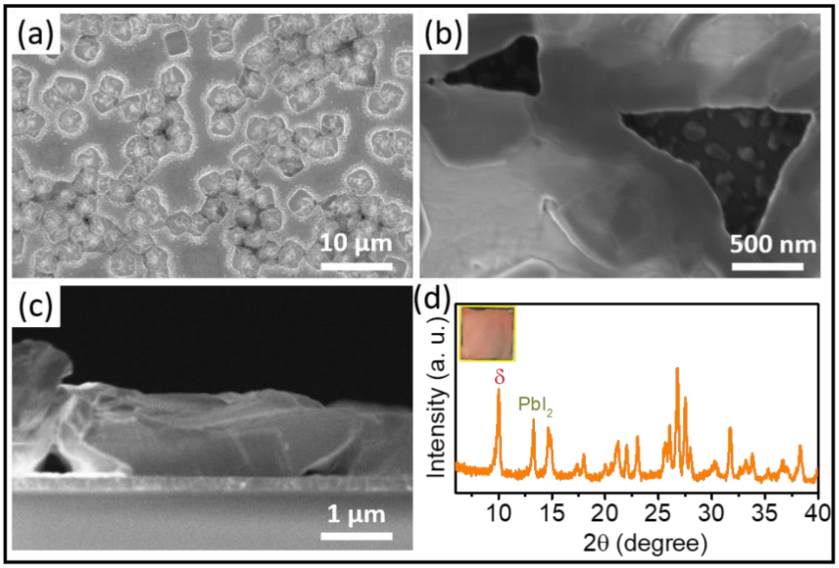

Figure S1. (a and b) surface and (c) cross-sectional SEM images, XRD pattern of resulted $CsPbI_2Br$ perovskite film of directly annealed wet precursor film (spin-coated for 30 s) at160 ^{o}C without gradient steps.

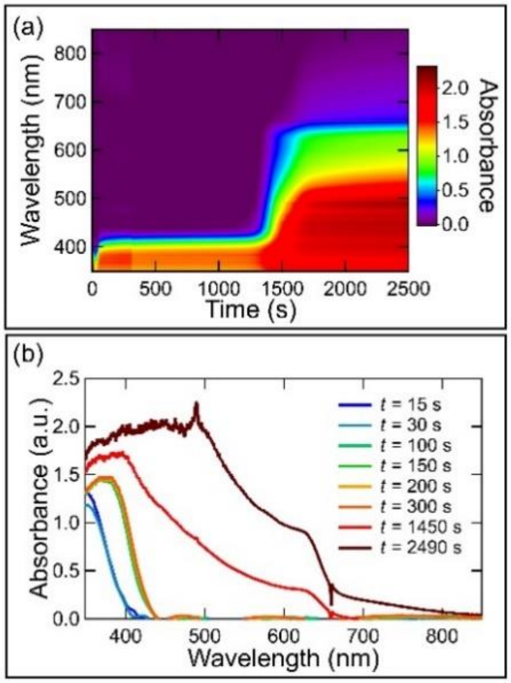

Figure S2. *In situ* UV-vis absorbance measurements of the SF-perovskite films during spin-coating (0 s – 200 s) and drying (200 s – 2500 s). A contour plot of the film formation evolution, and line-scans at selected points in time are shown in (a) and (b), respectively.

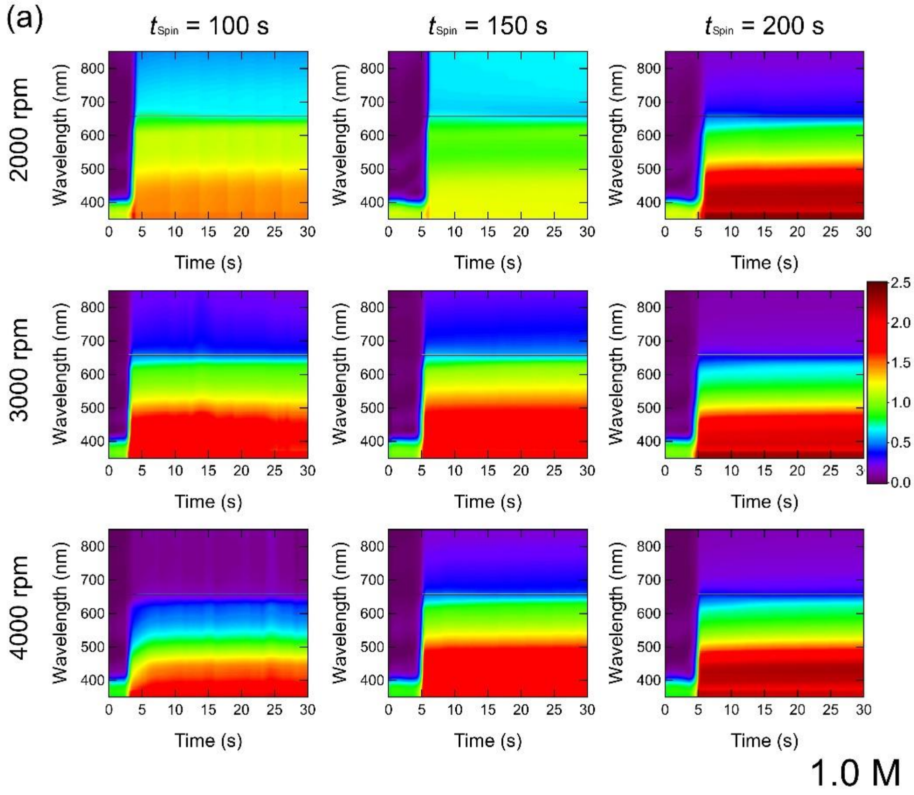

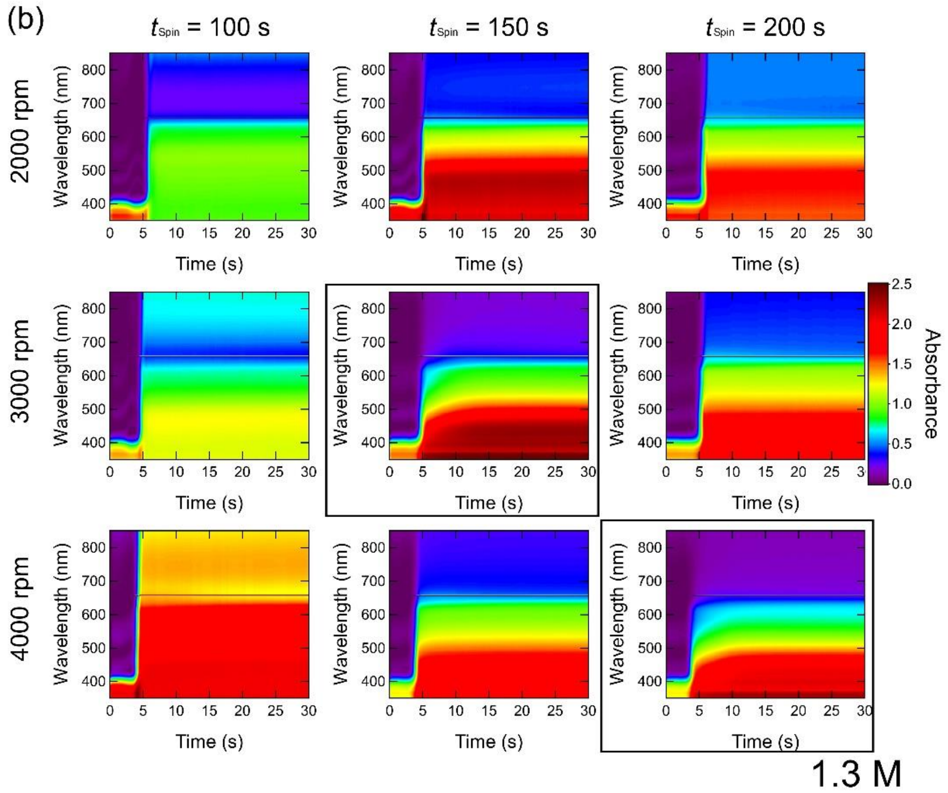
(b)
t_{Spin} = 100 s
t_{Spin} = 150 s
t_{Spin} = 200 s
2000 rpm
3000 rpm
4000 rpm
Wavelength (nm)
Time (s)
Absorbance
1.3 M

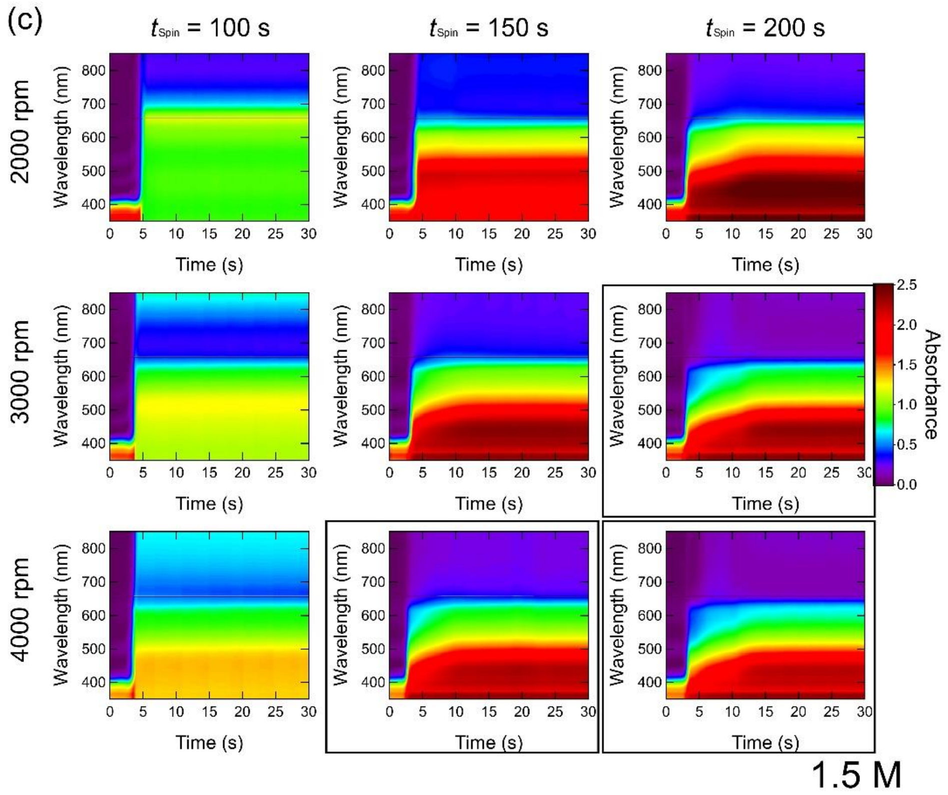
(c)
t_{Spin} = 100 s
t_{Spin} = 150 s
t_{Spin} = 200 s
2000 rpm
3000 rpm
4000 rpm
Wavelength (nm)
Time (s)
Absorbance
1.5 M

Figure S3. *In situ* measurements of the absorbance during annealing of an IP-film following the SF method. Panels (a), (b), and (c) show data obtained with precursor concentrations of 1.0 M (a), 1.3 M (b), and 1.5 M (c). We varied the duration of the spin-coating (columns) and the spin-rate (lines) for each concentration. Conditions that lead to an optimal evolution of the absorbance edge were selected (and marked by a black frame) according to the criteria that were found in the main article: The presence of the intermediate phase IIb and a sharp absorbance edge.

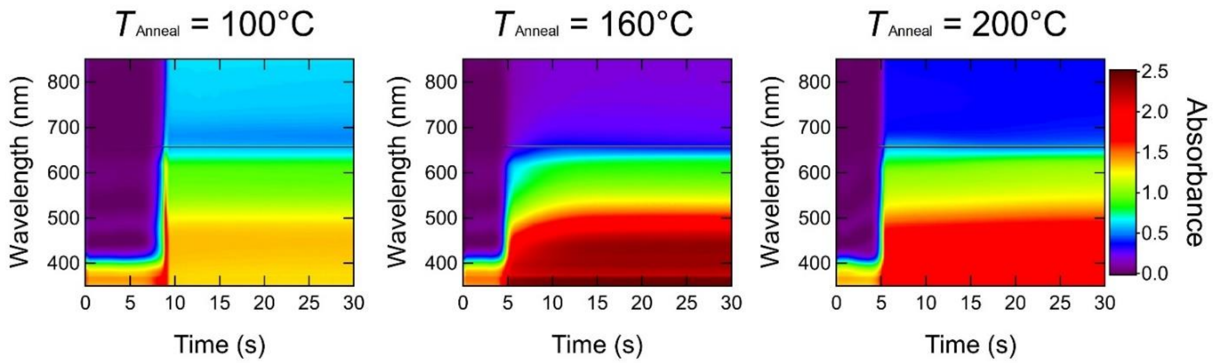

Figure S4. *In situ* measurements of the absorbance during annealing at varying temperature (T_{Anneal}) of an IP-film following the SF method.

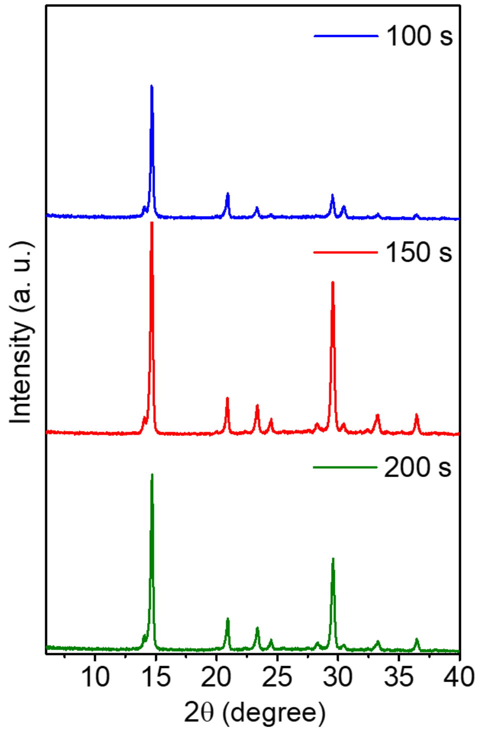

Figure S5. XRD patterns of fabricated perovskite films from (a) 100 s, (b) 150 sand (c) 200 s spin-coating of perovskite precursor on substrates.

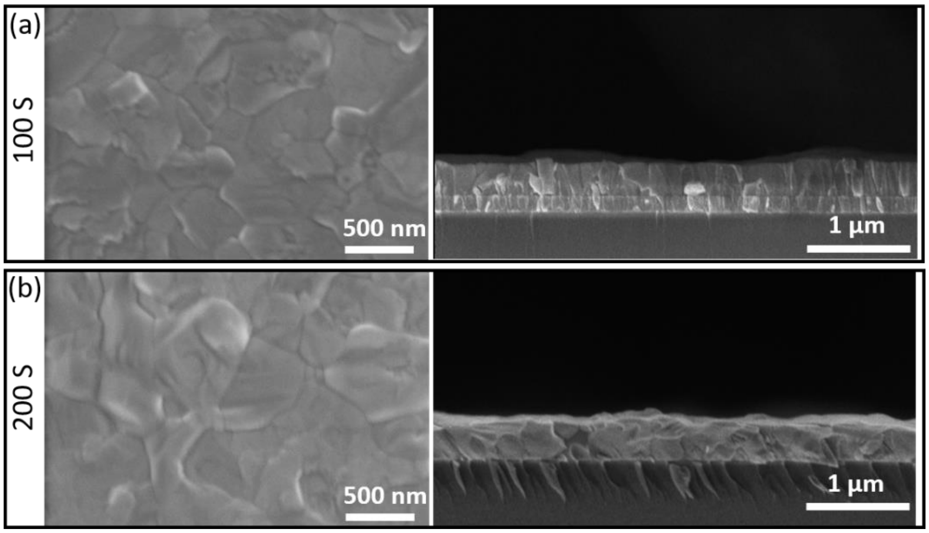

Figure S6. Top and cross-sectional SEM images of (a) SF-100 and (b) SF-200 films.

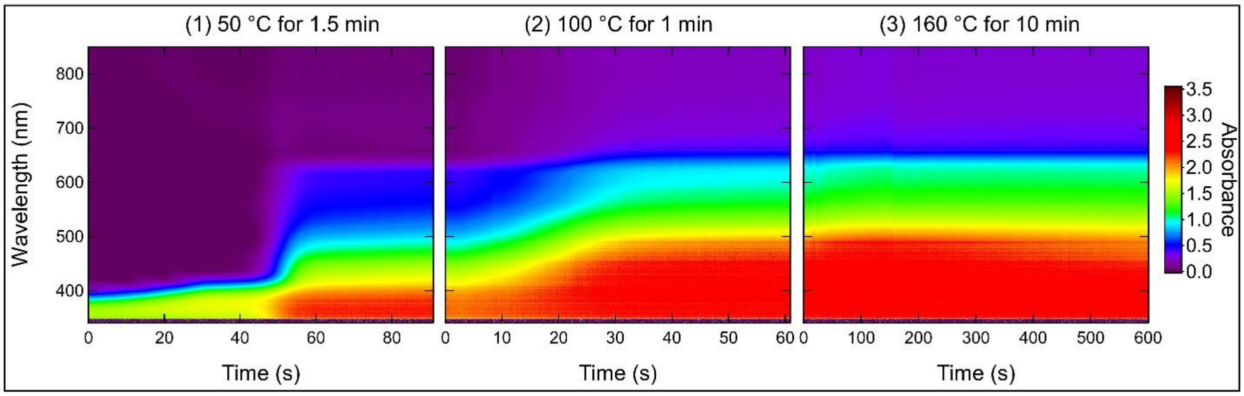

Figure S7. *In situ* measurements of the absorbance during annealing of an IP-film following the TG method. Each panel shows one of the three annealing steps. Note that three different samples were used to record these spectra.

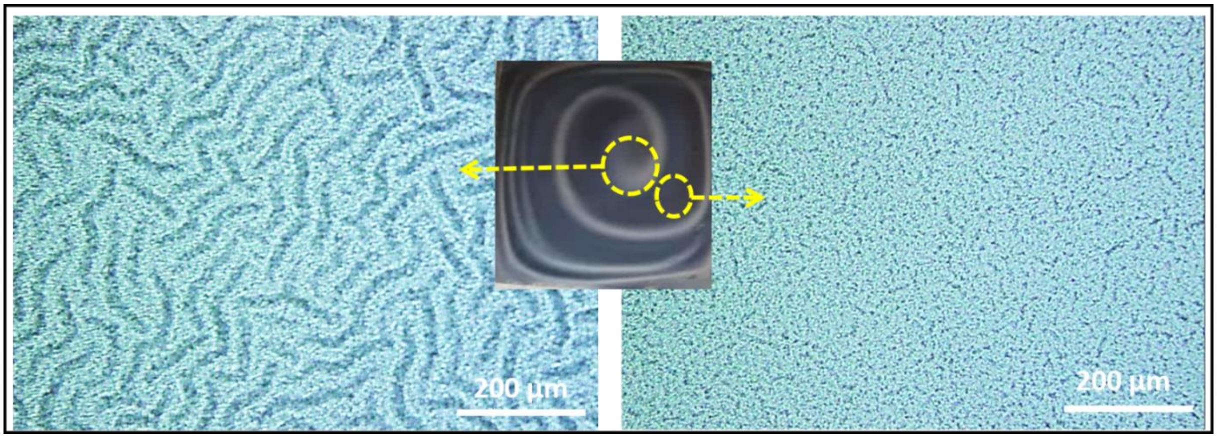

Figure S8. The achieved OM images of fabricated perovskite film by the TG method. Inserts: photo of the TG-film.

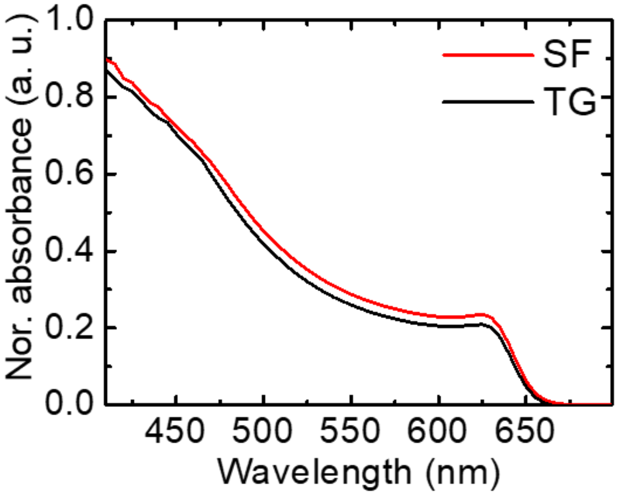

Figure S9. UV–vis absorbance spectra of SF and TG perovskite films.

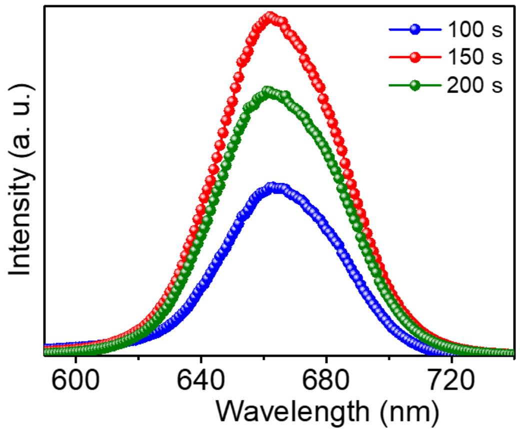

Figure S10. The SS-PL spectra of SF-films with different spin-coating times.

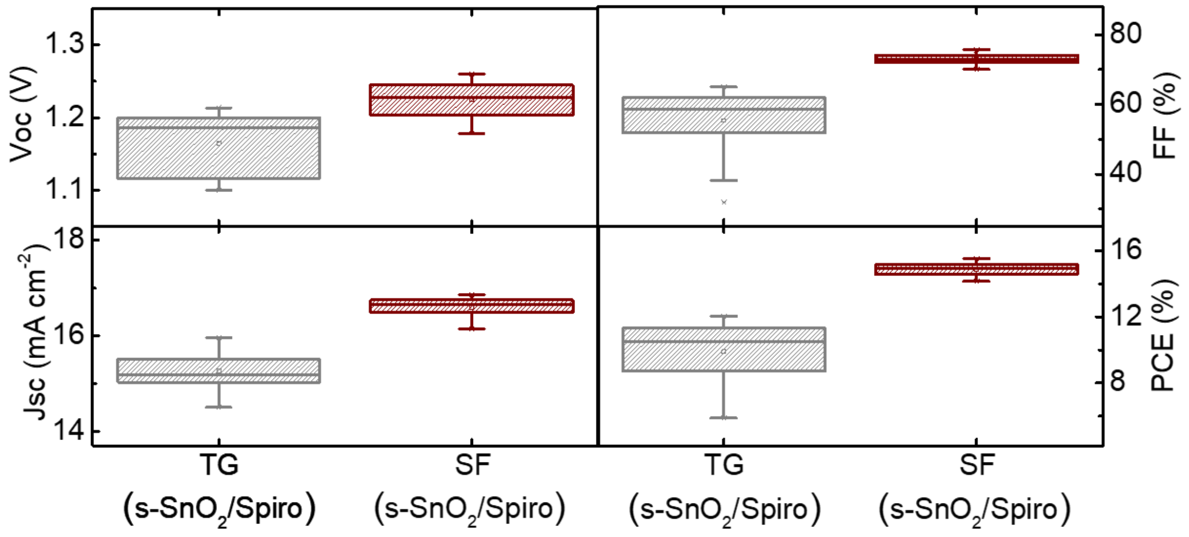

Figure S11. Photovoltaic parameters (V_{OC}, FF, $J_{SC,}$ and PCE) extracted from *J–V* measurement of 30 PSCs fabricated with TG- and SF-films (s-SnO_2 as ETL and Spiro as HTL).

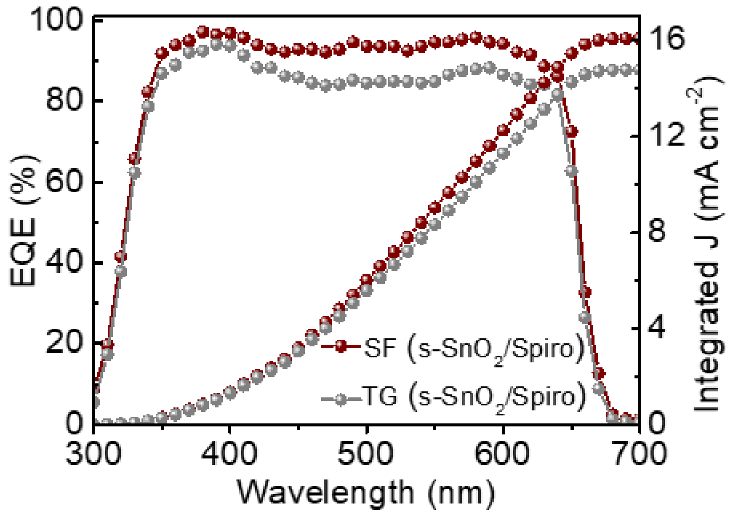

Figure S12. External quantum efficiency (EQE) spectra and the calculated J_{SC} of TG and SF-PSCs (s-SnO_2 as ETL and Spiro as HTL).

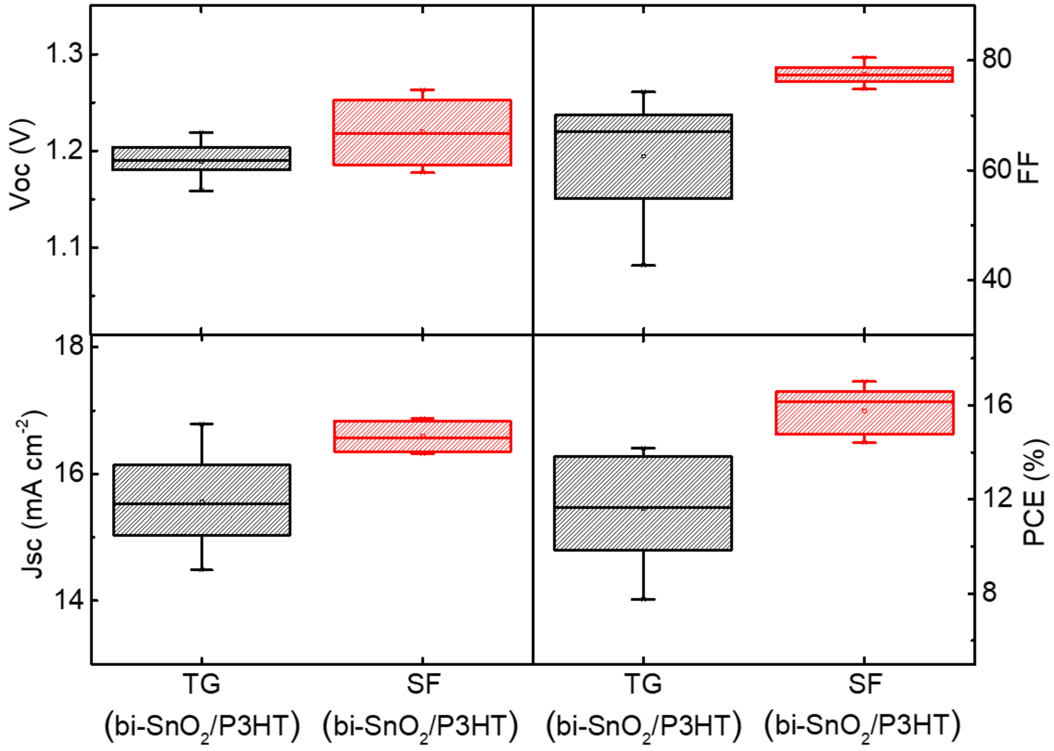

Figure S13. Photovoltaic parameters (V_{OC}, FF, $J_{SC,}$ and PCE) extracted from *J–V* measurement of fabricated PSCs with TG- and SF-films (bi-SnO_2 as ETL and P3HT as HTL).

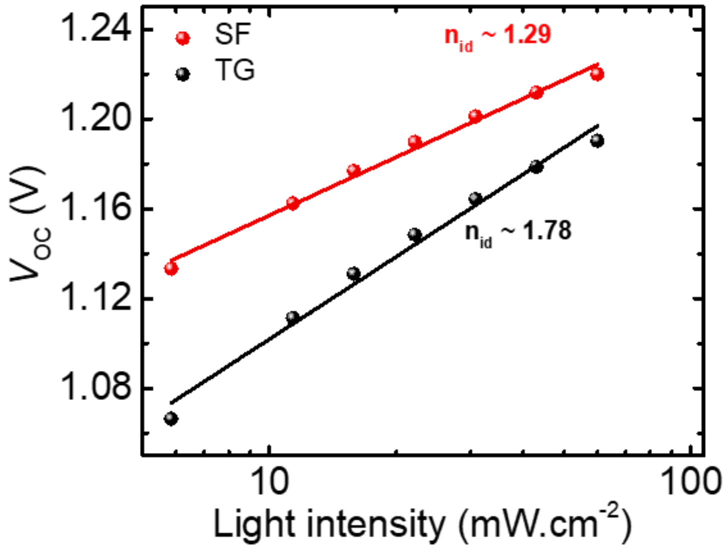

Figure S14. V_{OC} vs. light intensity measurements derived from TG and SF PSCs.

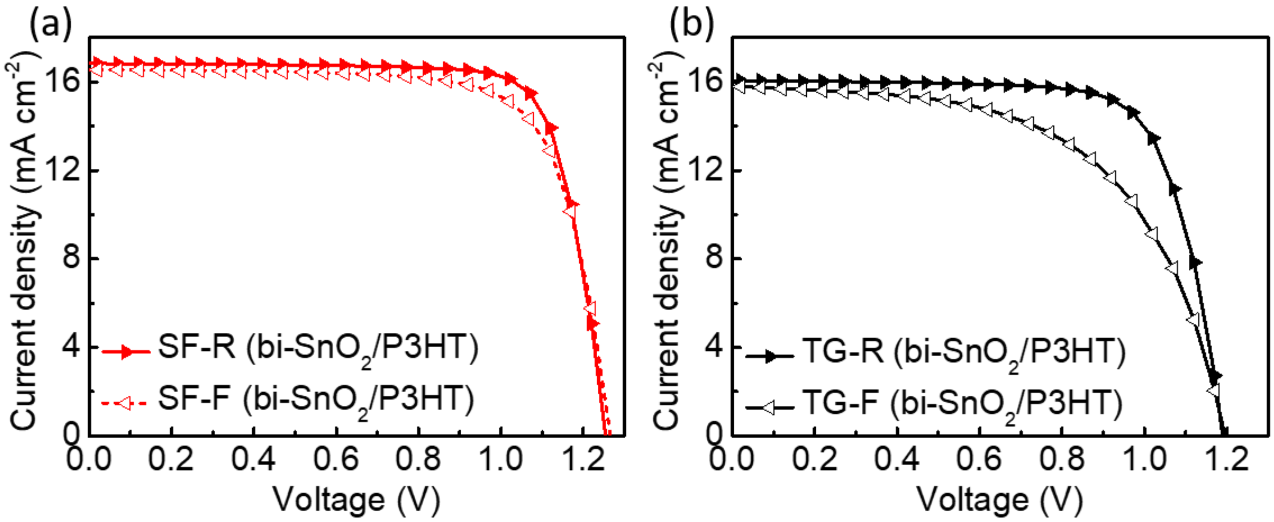

Figure S15. The reverse and forward scans *J–V* characteristics of (a) SF- and (b) TG-PSCs (bi-SnO_2 as ETL and P3HT as HTL).

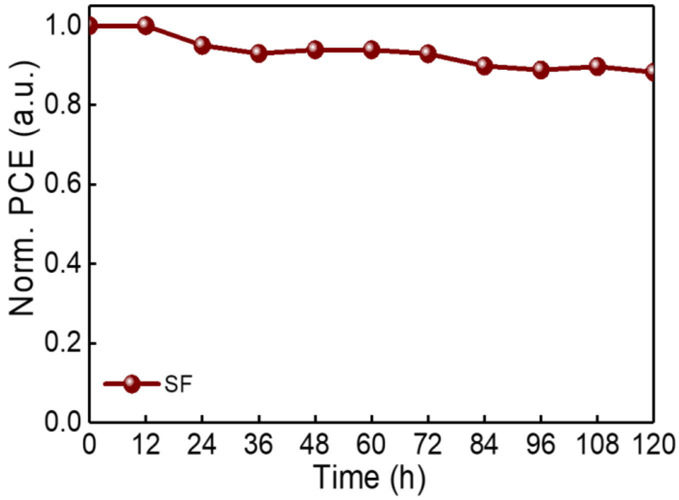

Figure S16. Thermal stability of devices at 85 ^oC under N_2 condition (average value of 6 solar cells).

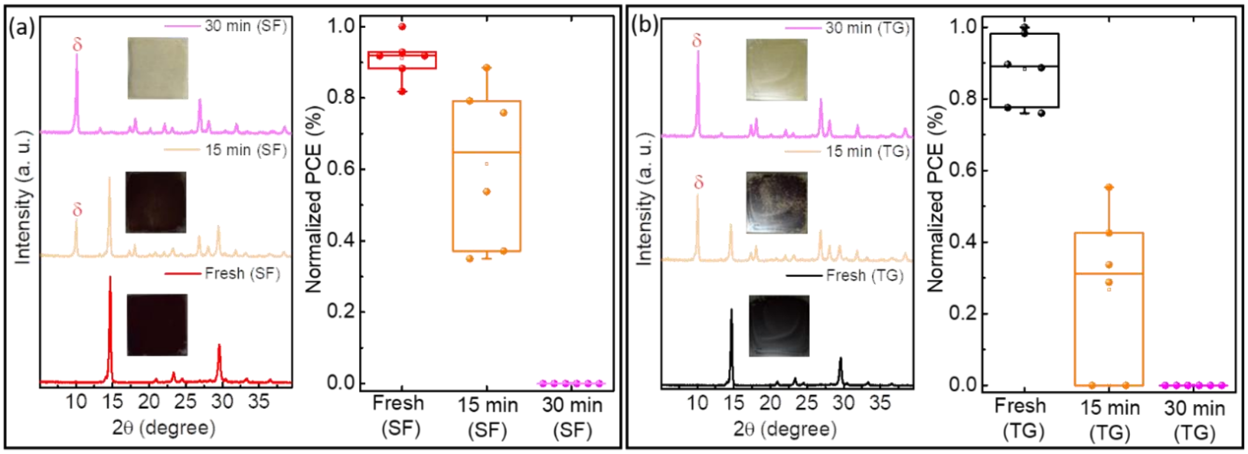

Figure S17. The XRD patterns and normalized *PCE*s of (a) SF- and (b) TG- films, before and after exposure to the high humidity of ~ 60 % for 15 min and 30 min. Insets: photos of the appearance of different perovskite films.

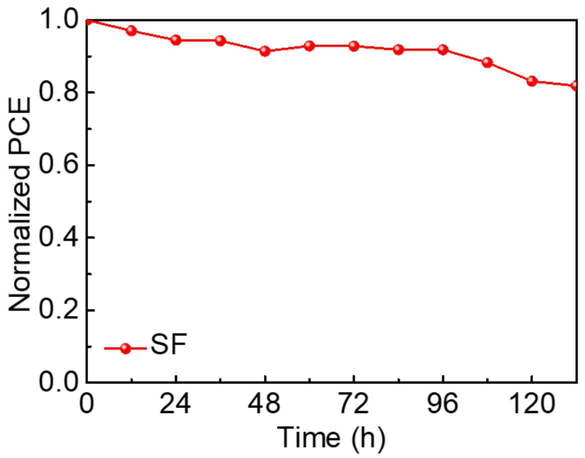

Figure S18. The UV stability of unencapsulated SF-PSCs under N_2 atmosphere (average value of 6 solar cells).

Table S1. Parameters of the TRPL spectroscopy based on different samples.

Samples	τ1 (ns)	τ2 (ns)	A1	A2
Glass/TG-film	1.9	8.2	0.19	0.33
Glass/SF-film	3.3	14.3	0.40	0.30

Table S2. Photovoltaic parameters obtained from the champion PSCs (under reverse and forward scans) fabricated by SF- and TG-films (bi-SnO_2 as ETL and P3HT as HTL).

PSCs	PCE (%)	J_{SC} (mA cm^{-2})	FF (%)	V_{OC} (V)
TG-R (bi-SnO_2/P3HT)	14.2	16.0	74.2	1.19
TG-F (bi-SnO_2/P3HT)	10.8	15.7	57.6	1.19
SF-R (bi-SnO_2/P3HT)	17.0	16.6	80.5	1.27
SF-F (bi-SnO_2/P3HT)	15.8	16.5	75.6	1.27

Table S3. The reported photovoltaic data for $CsPbI_2Br$ PSCs with over 16% PCEs.

Composition	Architecture	Voc (V)	Jsc (mA cm^{-2})	FF (%)	PCE (%)	Ref.
$CsPbI_2Br$	n-i-p planar	1.27	16.6	80.5	17.0	This work
$CsPbI_2Br$ (In-doped)	n-i-p mesoporous	1.33	16.3	80.1	17.4	[2] (world record)
$CsPbI_2Br$	n-i-p mesoporous	1.28	16.5	81.6	17.2	[3]
$CsPbI_2Br$ (Fe-doped)	n-i-p planar	1.31	15.9	81.8	17.1	[4]
$CsPbI_2Br$ (Nb-doped)	n-i-p mesoporous	1.31	16.2	77.0	16.4	[5]
$CsPbI_2Br$ (Ca-doped)	n-i-p mesoporous	1.32	15.3	83.2	16.7	[6]
$CsPbI_2Br$ (Cu-doped)	n-i-p planar	1.18	16.9	80.0	16.1	[7]
$CsPbI_2Br$	n-i-p planar	1.24	16.5	82.1	16.8	[8]
$CsPbI_2Br$	n-i-p planar	1.27	15.9	79.0	16.0	[9]
$CsPbI_2Br$	n-i-p planar	1.31	15.8	78.0	16.0	[10]
$CsPbI_2Br$	n-i-p planar	1.24	16.2	82.0	16.4	[11]
$CsPbI_2Br$	n-i-p planar	1.23	16.8	77.8	16.0	[12]

References:

[1] C. M. Sutter-Fella, *Adv. Energy Mater.* **2021**, *11*, 2003534.

[2] S. S. Mali, J. V Patil, P. S. Shinde, G. de Miguel, C. K. Hong, *Matter* **2021**, *4*, 635.

[3] S. Yang, J. Wen, Z. Liu, Y. Che, J. Xu, J. Wang, D. Xu, N. Yuan, J. Ding, Y. Duan, S. (Frank) Liu, *Adv. Energy Mater.* **2022**, *12*, 2103019.

[4] T. Ozturk, E. Akman, A. E. Shalan, S. Akin, *Nano Energy* **2021**, *87*, 106157.

[5] J. V Patil, S. S. Mali, C. K. Hong, *ACS Appl. Mater. Interfaces* **2020**, *12*, 27176.

[6] Y. Han, H. Zhao, C. Duan, S. Yang, Z. Yang, Z. Liu, S. (Frank) Liu, *Adv. Funct. Mater.* **2020**, *30*, 1909972.

[7] K.-L. Wang, R. Wang, Z.-K. Wang, M. Li, Y. Zhang, H. Ma, L.-S. Liao, Y. Yang, *Nano Lett.* **2019**, *19*, 5176.

[8] P. Wang, H. Wang, Y. Mao, H. Zhang, F. Ye, D. Liu, T. Wang, *Adv. Sci.* **2020**, *7*, 2000421.

[9] W. Tang, Y. Chen, J. Yang, R. Yuan, Y. Lv, Q. Ma, Y. Wu, P. Zhang, W.-H. Zhang, *J. Power Sources* **2021**, *482*, 228965.

[10] J. Li, J. Yang, J. Ma, J. Liang, Y. Liu, X. Hu, C. Chen, W. Yang, J. Min, Q. Bao, G. Fang, C. Tao, *Chem. Eng. J.* **2021**, *417*, 129247.

[11] X. Li, W. Chen, S. Wang, G. Xu, S. Liu, Y. Li, Y. Li, *Adv. Funct. Mater.* **2021**, *31*, 2010696.

[12] W. Chen, H. Chen, G. Xu, R. Xue, S. Wang, Y. Li, Y. Li, *Joule* **2019**, *3*, 191.
